# Cancer Drug Sensitivity Prediction Based on Deep Transfer Learning

**DOI:** 10.3390/ijms26062468

**Published:** 2025-03-10

**Authors:** Weijun Meng, Xinyu Xu, Zhichao Xiao, Lin Gao, Liang Yu

**Affiliations:** 1 School of Computer Science and Technology, Xi’an University of Posts & Telecommunications, Xi’an 710071, China; mengweijun@xupt.edu.cn; 2 School of Computer Science and Technology, Xidian University, Xi’an 710071, China; 22031212455@stu.xidian.edu.cn (X.X.); x1425906650@163.com (Z.X.); lgao@mail.xidian.edu.cn (L.G.)

**Keywords:** deep transfer learning, domain-adapted approach, drug sensitivity, multi-source data

## Abstract

In recent years, many approved drugs have been discovered using phenotypic screening, which elaborates the exact mechanisms of action or molecular targets of drugs. Drug susceptibility prediction is an important type of phenotypic screening. Large-scale pharmacogenomics studies have provided us with large amounts of drug sensitivity data. By analyzing these data using computational methods, we can effectively build models to predict drug susceptibility. However, due to the differences in data distribution among databases, researchers cannot directly utilize data from multiple sources. In this study, we propose a deep transfer learning model. We integrate the genomic characterization of cancer cell lines with chemical information on compounds, combined with the Encyclopedia of Cancer Cell Lines (CCLE) and the Genomics of Cancer Drug Sensitivity (GDSC) datasets, through a domain-adapted approach and predict the half-maximal inhibitory concentrations (IC50 values). Afterward, the validity of the prediction results of our model is verified. This study effectively addresses the challenge of cross-database distribution discrepancies in drug sensitivity prediction by integrating multi-source heterogeneous data and constructing a deep transfer learning model. This model serves as a reliable computational tool for precision drug development. Its widespread application can facilitate the optimization of therapeutic strategies in personalized medicine while also providing technical support for high-throughput drug screening and the discovery of new drug targets.

## 1. Introduction

Pharmaceutical research, as we know it today, began when chemistry reached a threshold level of maturity, guided by pharmacology and clinical sciences, and has contributed to the advancement of medicine more than any other scientific factor [1]. The emergence of molecular biology, especially the development of genomic science, has had a profound impact on drug discovery [2,3,4]. Genome science, combined with bioinformatics tools, enables us to identify the genetic bases of multifactorial diseases to select the most appropriate treatments [5,6]. Molecular biology enables the understanding of disease processes at the genetic level and the identification of optimal molecular targets for drug intervention [7,8,9]. Target modification is the highest level of validation for drug discovery, such as the blockade of receptors or the inhibition of enzymes under the action of a drug, resulting in reversal of the disease state. Whereas phenotypic changes in isolated cells caused by compounds that modify a target constitute a minimal validation, if the phenotypic changes can be reproducibly induced in animal models of certain disease-related mechanisms and the confidence of the target varies with the animal model as the number increases, the modification of that target results in the desired phenotypic change [10,11,12].

Drug discovery is mainly based on molecular target discovery and phenotypic screening, the former being the main method of drug discovery in recent decades. However, in recent years, most of the large number of approved drugs originated from phenotypic screening, and the exact mechanism of action or molecular target of each was elaborated. High-throughput screening techniques have provided diverse drug susceptibility data for cancer cell lines and hundreds of compounds, and large-scale pharmacogenomics studies of cancer genomes have provided unprecedented insights into the study of anticancer therapies to determine putative predictions of drug susceptibility and to conduct phenotypic screening to develop new anticancer drugs for anticancer therapy [13]. The Cancer Cell Line Encyclopedia (CCLE) [14] and the Genomics of Drug Sensitivity in Cancer (GDSC) [15] datasets are the most popular datasets in this field.

Currently, many research groups have used computational methods for drug sensitivity prediction, most of which are based on machine learning. The simultaneous modeling of compound and cell line characteristics facilitates drug sensitivity prediction, drug side effect analysis, and model extrapolation to new compounds and cell lines [16]. Menden et al. integrated cell line genomic features, including microsatellite sequences, sequence and copy number variations, and one-dimensional (1D) and two-dimensional (2D) features of compounds. A model for predicting the half-maximal inhibitory concentration (IC50) was established by using a neural network and Random Forest (RF) [17]. Another study built a dual-layer integrated cell line-drug network containing a cell line similarity network and a drug similarity network based on the Pearson correlation coefficients of the gene expression profiles of the cell line and information on compounds for predicting IC50 values [18]. Studies have also used multitask learning to integrate various omics data to predict drug responses [19,20]. However, drugs and cell lines are often represented by predefined features, such as structural features and omics features, respectively. Traditional machine learning-based methods often face the “small n, large p” problem. This is because the number of cell lines is much smaller than the number of genes in the gene map, which limits the prediction performance of traditional machine learning-based methods. As a pivotal branch of machine learning, deep learning leverages the construction of multi-layer nonlinear neural network architectures to autonomously learn hierarchical abstract features from data, thereby enabling the identification and prediction of complex patterns through these high-level representations [21,22,23,24,25]. In studying drug responses, various studies have used cell line genomic features and the structural features of drugs to predict drug susceptibility. DeepDR [26] is a model for predicting IC50 values with deep neural networks. In DeepDSC [27], a stacked autoencoder is used to extract genomic features of cell lines, and these features are combined with drug fingerprints to predict IC50 values. Drug fingerprints refer to distinctive chemical profiles or spectral patterns utilized for the identification and differentiation of pharmaceutical compounds, which are extensively applied in drug analysis. For instance, the infrared spectroscopy or mass spectrometry profiles of specific drugs can serve as unique fingerprints to facilitate the precise identification of their chemical constituents. In GraphDRP [28], the molecular structure of a drug is converted into a graph structure and then a graph convolutional neural network is used to obtain a representation of the drug, which is combined with a one-dimensional convolutional representation of the copy number variation feature of the cell line to predict the drug response.

Transfer learning is a machine learning approach that enhances learning efficiency and performance on related but distinct tasks by leveraging knowledge gained from one task. Transfer learning methods can be applied to datasets that are in the same feature space but have different distributions. However, in transfer learning, we need to assume that there is some consistency between the datasets. According to HaibeKains et al. [29], the GDSC and CCLE databases have a good correlation in terms of gene expression [30]. This implies that the gene expression data in these two databases have a certain level of consistency and similarity within the same feature space. Such consistency provides the foundation for transfer learning, as it suggests that the patterns learned from one database regarding the relationship between gene expression and drug response may be applicable to another database as well. The database researchers noted that the drug response data mostly corresponded to drug-insensitive lines with far fewer outliers and speculated that biological differences between the cell lines contributed to the poorer correlations. By applying the knowledge learned about the relationship between gene expression and drug response from one database to another, the overlapping information can be leveraged to enhance the accuracy of drug response models. There is significant overlap between the GDSC and CCLE databases, and both databases provide information on the same biological processes, making them suitable candidates for transfer learning methods that can be used to further design more accurate drug response models. Saugato et al. [31] proposed two classes of transfer learning solutions for drug response prediction. One is a transfer learning method based on cost optimization, which mainly establishes a relationship between the two latent variables, namely, the GDSC and CCLE database gene expression values, and the AUC (area under the dose—response curve) values. The other is the domain adaptation method, which looks for a mapping relationship between the gene expression values and AUC values. This method considers the mapping of the gene expression values between the two databases to be linear and the mapping of the AUC-predicted values to be nonlinear.

However, the number of cell lines in this task is much smaller than the number of genetic maps, which limits the predictive performance of traditional machine learning-based methods. Deep learning has been widely used to extract features from complex, high-dimensional features and complete target prediction tasks, and its performance is even better. Existing drug sensitivity methods based on deep learning often incorporate numerous features of cancer cell lines, such as sequence variations, copy number variations, etc. However, these features are not all easily available for different cancer cell lines. All physiological manifestations of organisms are derived from the most basic gene expression. Therefore, one of the motivations for this work is to mine the key features of cancer cell line gene expression values for prediction tasks based on deep feedforward networks. Inspired by deep learning and transfer learning, this paper proposes a deep transfer learning framework that integrates the gene expression profiles of two database cell lines and the molecular structures of compounds. This method is called DADSP (Domain Adaptation for Drug Sensitivity Prediction). Our model extracts the features of gene expression maps from stacked self-encodings, obtains low-dimensional representations of these features, and combines them with molecular features of compounds to obtain prediction results through a deep feedforward network. Combined with our validity verification, it is confirmed that our model conforms to the biological mechanism of drug response. Hence, it is not only a supplement to deep learning in drug response prediction but also provides a means for transferring data distribution processing among different databases of the same biological process.

The codes and datasets are available at https://github.com/xzc196/DADSP, (accessed on 5 March 2025).

## 2. Results

### 2.1. Performance Comparison with Other Algorithms

To comprehensively evaluate the performance of the DADSP method, this section compares the DADSP method with commonly used domestic and international methods. We refer to the model structure of [10], remove the feature extractor and regression predictor of the model separately, and keep the rest of the parts consistent to form a standard deep feedforward network drug susceptibility prediction model. The comparison is primarily based on ablation experiments to verify the effectiveness of the transfer learning module in the model. In these experiments, the drug features are all represented using the Morgan fingerprint. The following models are introduced in the ablation experiments: DADSPA-: a model without pre-training using a stacked autoencoder. DeepDSC-1 [27]: a deep feedforward network model that does not use a domain adversarial discriminator and is trained and tested only on target domain data. DeepDSC-2 [27]: a model with the same network structure as DeepDSC-1, but the parameters are transferred using a stacked autoencoder trained on both source and target domain data. SLA (Selective Learning Algorithm) [32]: a model that defines an intermediate domain to capture source domain information and transfer it to the target domain. In this model, the data from the GDSC and CCLE are jointly used to pre-train a stacked autoencoder, and the parameters are then transferred to a feedforward neural network. Additionally, different choices of regression predictors were also compared, including Random Forest (RF) [33], Logistic Regression (LR) [34], and Support Vector Regression (SVR) [35], as shown in Table 1.

From Table 1, we can see that the DeepDSC-1 model, which only uses target domain data, performs poorly, indicating the necessity of transfer learning in this work. The *RMSE* difference between the DADSPA- model without pre-training and the DADSP-A model is 0.6, demonstrating the important contribution of the stacked autoencoder to the genomic representation. The traditional machine learning methods LR, RF, and SVR do not perform as well as the feedforward neural network, reflecting the training advantage of neural networks on large-scale data. In terms of transfer learning strategies, the adversarial-based DADSP-A method outperforms the domain difference and intermediate domain-based DADSP-B and SLA methods. This paper hypothesizes that for the feature representation of drug sensitivity data, the adversarial-based method, which uses a feedforward neural network binary classifier as the main implementation of transfer learning, is more effective in extracting nonlinear representations between domains, and therefore performs better.

### 2.2. Blind Test

In a normal test experiment, a drug/cell line appears in both the training and test sets, although its drug response data are not involved in training. However, predicting responses to unseen drugs/cell lines is more challenging. Therefore, in blind testing experiments, the drugs/cell lines in the testing phase do not exist in the training phase, and we randomly delete a portion of the drugs/cell lines. Instead of removing drugs or cell lines separately for two blinded experiments, we opted to remove both drugs and cell lines simultaneously, making the model predictions more challenging. Our blind test results for different models are presented in Table 2.

As shown in Table 2, the model performance declined across the board compared to the standard test results, which also confirms that the adversarial DADSP-A still achieved the best performance in the blind test. However, DADSP-B exhibited unexpectedly poor performance in the blind test, which is suspected to be related to the randomly deleted cancer cell lines or drug data, potentially requiring the setting of new hyperparameters to adapt to the training. The results of DeepDSC-1 and SLA did not differ significantly, and both transfer learning strategies achieved certain effects. The blind test experiment examined the model’s ability to explore the common hidden feature representations between different drug and cancer cell line characteristics. The adversarial-based method performed the best, which also indirectly confirms its unique advantage in domain adaptation feature distance.

### 2.3. Comparison of the Characteristics of Different Drugs

In addition to cell line gene expression features, the extraction methods that are used for drug feature representation have a huge impact on the drug sensitivity prediction task. We compare different extraction methods of drug molecular features. We use DADSP-A for experiments, only changing the drug feature extractor and leaving the rest of the model unchanged. We screen four drug feature extraction methods, all from the current state-of-the-art deep drug sensitivity prediction models. They are known as the hashed Morgan fingerprints of drug molecules [36], struct similarity profiles (SSPs) based on user-defined reference drugs [37], two-dimensional matrix information of drug molecules extracted and constructed by convolutional neural networks (CNNs) [38], and drug molecular graph structures that are extracted using graph convolutional neural networks (GNNs) to obtain individual drug representations [28,39]. We compare the results, as presented in Table 3.

According to the results, the hashed Morgan fingerprints of drug molecules perform the best. A Morgan fingerprint hashes the molecular features of each layer and each atom into a bit vector. Each layer considers the atomic fingerprints that are less than the specified maximum distance from the starting atom and then diffuses out layer by layer. This approach can better express the molecular structure characteristics of the drug. Although convolutional neural networks and graph convolutional neural networks have powerful effects in feature extraction, we must first process the drug molecular structure into a corresponding two-dimensional matrix structure and graph structure defined by the user. The performance on this task may be limited by this.

### 2.4. Uknown Drug Response Prediction

**Figure 1:**
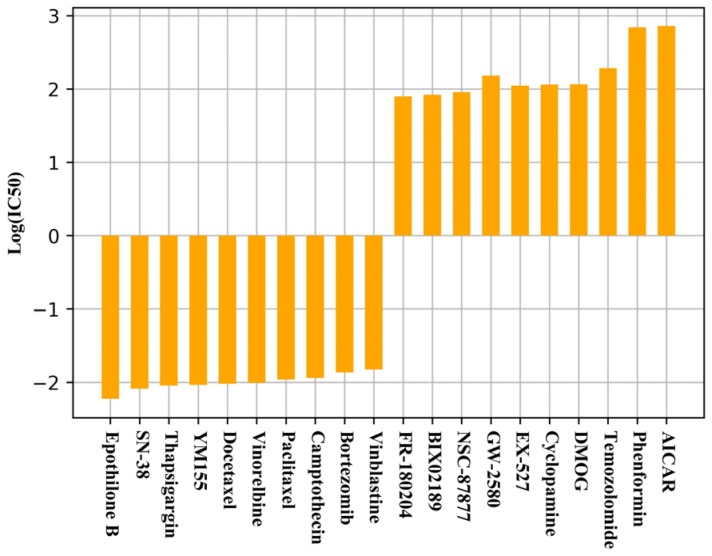
The 10 drugs with the lowest and highest log(IC50) values in the unknown drug response experiment.

In this part of the experiment, we use the best performing model in the normal experiment to predict the IC50 values of a missing pair from a dataset of 39,000 missing drug response pairs that we screened. Figure 1 shows the predicted top and bottom 10 drugs. The results show that, in this part of the experiment, we predict the lowest IC50 for Epthilone B, which can organize cell division by interacting with microscopic proteins [40], and the remaining nine drugs all play positive roles in the treatment or inhibition of cancer. Meanwhile, AICA R and phenformin are predicted to have the highest IC50 values, implying that the cancer is not sensitive to these two drugs. AICA R is used clinically to treat and prevent ischemic heart damage [41], while phenformin is used by people to treat diabetes [38]. Our prediction results regarding the two drugs with the highest IC50 values are the same as those in [28], and the 10 drugs with the lowest IC50 values also have multiple overlaps.

This evidence suggests that cancers are less sensitive to drugs with high IC50 values and that our model is effective in predicting missing pairs of potential drug responses.

### 2.5. Predicting Critical Genes for Drug Responsiveness

**Figure 2:**
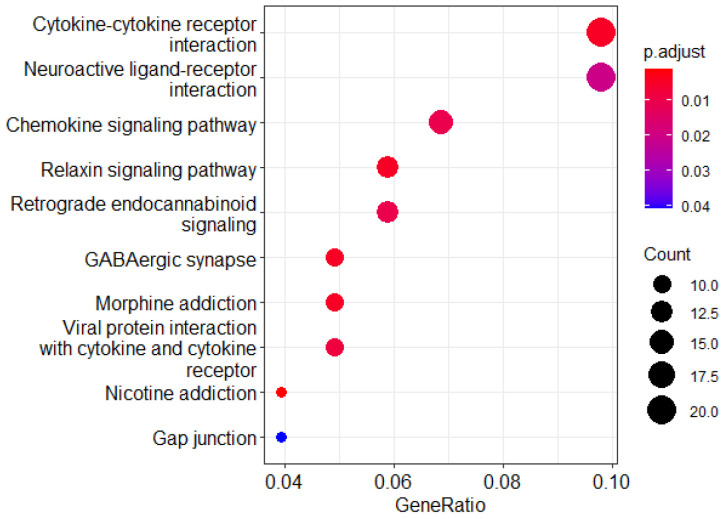
Pathways enriched for key genes in ZR-75-30 cells. Multiple pathways are involved in the mechanism of action or pathogenesis of cancer.

Deep learning models can extract expression information from input data but suffer from poor interpretability. While our model makes good predictions, an understanding of the genetic signatures that are active in the model is also necessary. Therefore, this experiment selects the most sensitive drug, Epothilone B (EpoB), and the three cell lines with the lowest IC50 values for this drug to further study the contributions of genetic characteristics to the prediction of the cell line response. At the same time, we introduce the integrated gradient method [42], which is a state-of-the-art feature interpretation method for deep neural networks. This method can feed back the contributions of active genetic features to the model to the input layer, and then key genes can be obtained. Specifically, key genes are identified based on the accumulation of neuron gradients along the path of the fully connected layer in the neural network. Table 4 presents the ten genes with the highest scores in the three cell lines. Metallothionein 1M in the MNK7 and ZR-75-30 cell lines is predicted to be the most critical gene. The high expression of this gene in gastric cancer tissue is a promoting factor of gastric cancer invasion and metastasis and is related to the occurrence of gastric cancer [43,44]. At the same time, it plays an important role in the hormone regulation of breast cancer and the occurrence of breast cancer. We also perform KEGG gene enrichment analysis on the top few hundred key genes of the ZR-75-30 cell line using the R package ClusterProfiler, version 4.14.6, https://www.bioconductor.org/packages/release/bioc/html/clusterProfiler.html, (accessed on 5 March 2025) [45], and the results are shown in Figure 2.

The three pathways with the largest numbers of critical genes, namely, the cytokine–cytokine receptor interaction, neuroactive ligand–receptor interaction [46], and chemokine signaling pathways, are all related to the pathogenesis of breast cancer [47,48,49]. Epothilone has been reported to be a nontaxane microtubule stabilizer that has a similar mode of action to paclitaxel but is active in paclitaxel-resistant cells. Metallothionein is highly expressed in the response of EpoB to cancer and may play a role in chemoresistance [50].

According to the results, our deep learning model can accurately extract the active genetic features in the drug response, which is in line with the antitumor mechanism of the drug.

### 2.6. Feature Space Comparison After Domain Adaptation

**Figure 3:**
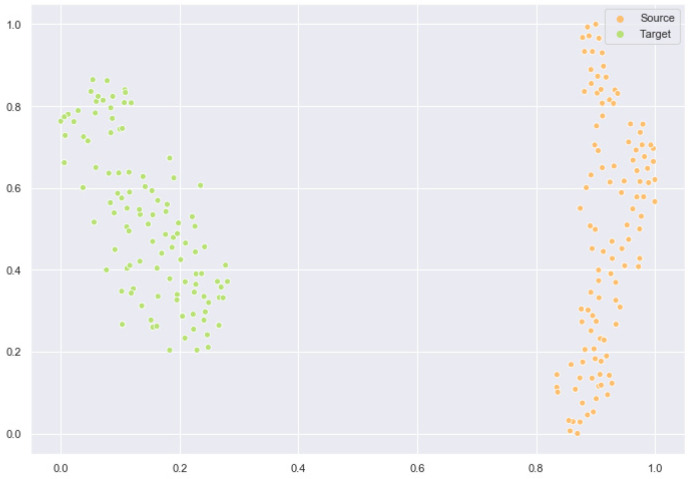
Feature space before domain adaptation.

**Figure 4:**
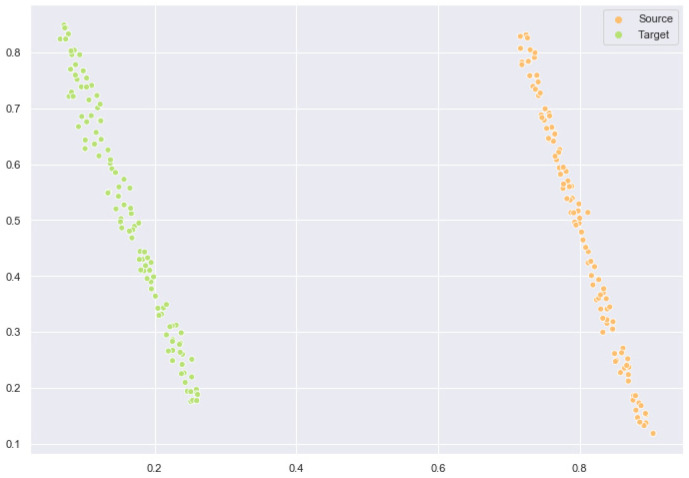
Feature space after domain adaptation.

Next, we conduct a comparison experiment of the feature space before and after model training. We use the t-SNE [51] algorithm to map high-dimensional features into a two-dimensional space. Figure 3 shows the feature space of the initial source and target domains. Figure 4 shows the feature space after we trained the model. As shown in the figure, the data feature distributions of the source domain and the target domain after domain adaptation are significantly closer. A similar data distribution ensures the prediction effect of the test set.

Overall, our domain adaptation algorithm achieves the expected results and plays an important role in the post-transfer tests.

## 3. Discussion

This article proposes a domain-adaptive drug sensitivity prediction method called DADSP and provides a detailed description. The DADSP method takes cancer cell line gene expression data, drug feature data, and drug sensitivity response data as the input and outputs the predicted drug sensitivity value IC50. Furthermore, this article provides a detailed explanation of the sources and specific forms of the input data. We elaborate on the specific model framework of the DADSP model, considering domain adaptation for different types of learning methods. We propose adversarial-based and domain discrepancy-based deep learning frameworks for drug sensitivity prediction tasks. In the model framework, we adapt the network parameters trained through unsupervised training of stacked autoencoders, which effectively reduce dimensionality and represent features well for high-dimensional features. We also provide a detailed comparison of DADSP with other methods in terms of model performance and transfer learning strategies. The adversarial transfer learning approach leverages the idea of using GANs to learn the implicit relationship function between the source domain and the target domain [52]. This implicit relationship is learned through complex nonlinear transformations in the network, making it more adaptable to different tasks. Therefore, the adversarial-based DADSP model in this article achieves the best performance on the target domain test set. Additionally, various methods are analyzed and discussed.

The article conducts effective analysis of the model’s prediction results. Firstly, a comparative experiment is conducted on different drug feature extraction methods, and it is found that the molecular fingerprint-based extraction method is more suitable for feedforward neural networks. The active genetic features in drug sensitivity prediction tasks are analyzed using the integral gradient method [42]. Through a series of research investigations, it is discovered that the active genetic features are all related to the mechanisms of cancer. The changes in domain feature distances before and after model training are visualized and analyzed, providing readers with a more intuitive understanding of the effects of domain adaptation. Through a series of analysis experiments on the model, the DADSP model not only completes the drug sensitivity prediction task but also confirms the successful application of domain adaptation methods in the problem of inconsistent data distributions.

## 4. Materials and Methods

### 4.1. Data Sources

In this study, we use cell line expression data and drug response data from GDSC and CCLE, as well as compound structure files from the PubChem [53] database. The GDSC and CCLE contain omics data and drug response data for thousands of cell lines. The gene expression (transcriptome data) of a cell line represents the level of activity of a gene when a cell from that line is in a certain state. The omics data also indicate whether a genome has produced mutations and copy number variations. The drug response is an important indicator for measuring whether cells are inhibited under the action of a drug. Specifically, it is the IC50 value or AUC value of the drug required to inhibit half of the biological activity of the cell line.

The GDSC is the largest cell line drug sensitivity database. Through preprocessing, we obtained drug response data for more than 213 drugs on 914 cell lines and obtained the expressions of 24 drugs on 504 cell lines from the CCLE. We downloaded array gene expression data for thousands of cell lines from both databases separately and processed all of these data via robust multi-array average (RMA) normalization. To better evaluate our model, we retrieved 16,017 shared genes from both databases. Therefore, we obtained standardized two-dimensional gene expression matrices from the two databases, and the values in the matrix primarily fell in the range of 3 to 10. To evaluate the drug response data, we selected IC50 as a measurement index, with a lower IC50 value indicating that the cell line was more sensitive to the corresponding drug. We treated IC50 values as -log10IC50 values. Data for more than 200 drugs in canonical SMILES format [54] were downloaded from the PubChem database through the RDKit package, version 4.14.6, https://www.rdkit.org/docs/index.html, (accessed on 5 March 2025) [55] and processed into the desired expression formats, including 256-bit hashed Morgan fingerprints. A drug graph representation structure was designed using the atomic features of Deepchem [39] and the molecular graph construction mode of GraphDRP [28]. In this article, CCLE data is regarded as the target domain dataset, that is, the test set. The size of the source domain dataset is about 10 times the size of the target domain dataset.

### 4.2. Model Input Data

This section will introduce the data format for input to the DADSP model. The input data mainly consists of three parts:

(1) The gene expression profiles of cancer cell lines, represented as d, where 16,016 is the number of shared genes and N is the number of training samples in a batch.
(2) Drug features, represented as $x_{2}\in {\mathbb{R}}^{N\times 256}$, where 256 is the length of the hashed Morgan fingerprint. Subsequent chapters will conduct experimental analysis on different ways of representing drug features.
(3) Cancer cell line–drug sensitivity data, represented in the format [Cell Line ID, Drug ID, IC50 value].

### 4.3. Deep Transfer Learning and Autoencoder

**Figure 5:**
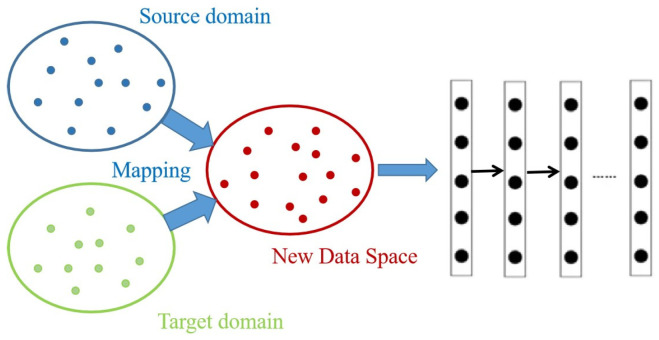
General framework for deep transfer learning. The source domain and the target domain are mapped into a common data space.

Deep learning has been used to process scRNA-seq data to extract an increasing number of abstract features of the original input through a series of nonlinearly transformed hidden layers in deep architecture learning [21]. In recent years, deep learning has achieved widespread application development in bioinformatics [56,57,58,59,60,61,62,63,64], computer vision [65,66], and natural language processing [67,68]. Deep transfer learning (DTL) is a technology that uses deep learning to build transfer learning models which has made great progress in the imaging field and includes methods such as mapping-based deep transfer methods [69] and adversarial-based transfer methods [70,71]. As illustrated in Figure 5, the data of the source domain and the target domain are mapped to a new data space to obtain new feature representations and then make predictions based on different task regressions or classifications. Domain adaptation [72,73] is an important method in deep transfer learning. It is often used in situations where the source domain data distribution is inconsistent with the target domain data distribution. Domain adaptation enables learners to generalize across different domains with different distributions by matching marginal and conditional distributions.

In this research, we use the GDSC database as the source domain and the CCLE database as the target domain, and we realize the transfer of knowledge from the GDSC to the CCLE. Before applying the domain adaptation strategy, we first extract high-level low-dimensional feature representations of the data through various methods.

**Figure 6:**
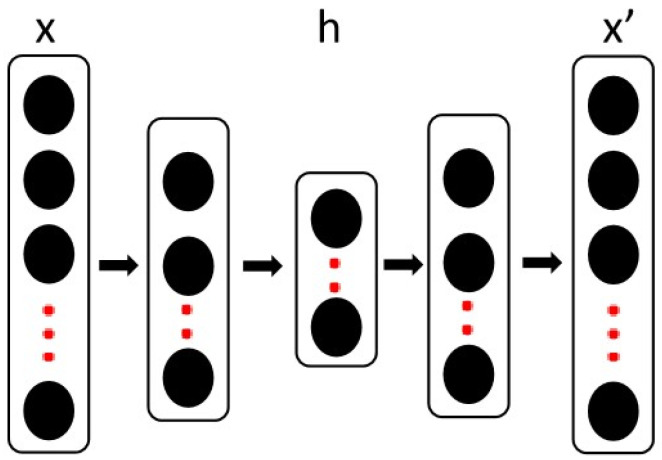
Architecture of a stacked autoencoder. *x* and *x*’ denote the input and the reconstructed output, respectively, and *h* denotes the encoded feature representation.

A stacked autoencoder (SAE) is a layer-by-layer unsupervised deep learning model that attempts to reconstruct the input under constraints such as low dimensionality and noise-free nature [74,75]. An SAE is a stack of multiple autoencoders, as illustrated in Figure 6, and the encoder and decoder are represented by Formulas (1) and (2).

(1) $$h=f_{\theta }(wx+b)$$

(2) $$x\prime =g_{\theta \prime }(h)=g_{\theta \prime }(f(wx+b))$$

where *x* is the input layer, *h* is the middle bottleneck layer, and *x*′ is the output layer after reconstruction.

During layer-by-layer training, a single self-encoding layer passes through a three-layer network x->h->x and the reconstruction loss between the original vector and the reconstructed vector is used for back propagation at the end of the training of this layer, the output layer is treated as a new input layer, and an autoencoder is trained for the next layer. Finally, the entire SAE network is fine-tuned. Low-dimensional representations are obtained by stacking the gene expression features of pretrained cell lines from autoencoders.

In this study, the test set is an independent target domain dataset, so in the unsupervised training, only the cancer genomic data from the source domain (GDSC database) is used. Finally, the encoder network parameters of the stacked autoencoder are used as the initial parameters of the DADSP gene feature extractor, which also applies the parameter fine-tuning transfer learning idea, saving the training time of the entire DADSP model.

### 4.4. Our Method

#### 4.4.1. Adversarial-Based Domain Adaptation Models

**Figure 7:**
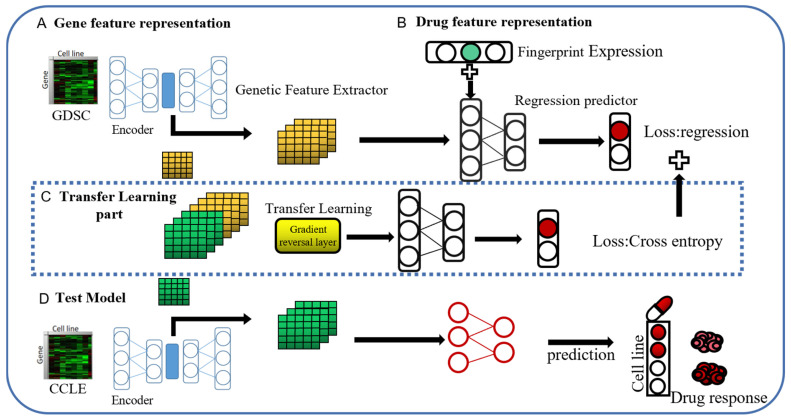
Flowchart of our DADSP-A, which consists of two feature extractors, a regression predictor, and a domain discriminator. (**A**): Gene feature representation, which uses an SAE to map high-dimensional gene expression to low-dimensional representations. (**B**): Drug feature representation. (**C**): Transfer learning part, which achieves the goal of feature proximity by maximizing the domain classification error. (**D**): Test module.

Deep feedforward networks are widely used in various fields in both regression tasks and classification tasks [76,77,78,79,80,81]. In the task of drug response prediction, deep feedforward networks exhibit strong modeling capabilities [26]. In deep learning drug susceptibility research, excellent performance has been realized on regression tasks and classification tasks [27,82,83,84,85]. According to [86], discrete drug response data category prediction involves information loss compared with regression tasks, so we propose a deep transfer learning model for predicting continuous drug sensitivity values (IC50 values). A flowchart of our DADSP A is shown in Figure 7. Our model can be divided into four parts, namely, a gene expression feature extractor, drug feature extractor, domain discriminator, and regression predictor. For the gene expression feature extractor, we first use a stacked autoencoder as the gene expression feature extractor to extract features from gene transcriptomes. This feature extraction module is composed of a feedforward neural network with the same structure as the encoder part of the stacked autoencoder mentioned earlier, and the parameters are initialized with the transferred network weights. During the training process, the source domain data and the target domain data are concatenated along the batch dimension and fed into this module jointly. For the drug feature extractor, we choose the hashed Morgan fingerprints of the compounds, which are represented as 256-dimensional vectors. We use a two-layer feedforward neural network to construct this feature extraction module. Next is the domain discriminator module, which is composed of a three-layer feedforward neural network. The input to this module is the feature vector from the last layer of the gene feature extractor. The source and target domain data concatenated along the batch dimension are then passed through the domain discriminator, which outputs a two-dimensional domain probability distribution. This is jointly optimized with the constructed domain labels of [0, 1] using the cross-entropy loss function. The expression of the cross-entropy loss function is

(3) $$L=-\left[y\log y^{\prime }+(1-y)\log(1-y^{\prime })\right]$$

where $y^{\prime }$ is the output of the domain discriminator and y is the true domain label. Based on the adversarial learning principle, we need to maximize this loss function to make the binary classifier unable to distinguish the source and target domain data, thereby achieving the goal of similar data distributions. While ensuring the prediction task, we also need to perform the domain adversarial objective task. Therefore, there is a gradient reversal layer (GRL) [70] in front of the domain discriminator module. The role of the GRL is to automatically reverse the gradients during backpropagation, while keeping them unchanged during forward propagation. The formula for the GRL is as follows:

(4) $$R_{\lambda }(x)=x$$

(5) $$\frac{dR_{\lambda }}{dx}=-\lambda I$$

(6) $$\lambda =\frac{2}{1+\exp\left(-\gamma \cdot p\right)}-1$$

where γ is a constant, set to 10, and *p* is the ratio of the current iteration number to the total iteration number.

Finally, we need to complete the regression prediction. The module consists of a four-layer feedforward neural network. During the training process, the hidden layer features from the gene feature extractor are split along the batch dimension to extract the source domain features, which are then concatenated with the drug features and fed into the network. The reason for not concatenating the drug features and gene expression features at the very beginning and inputting them into the deep feedforward network is that the scale of the drug features differs greatly from that of the gene features, and their feature contributions would be diminished.

The last layer of this module uses the Sigmoid activation function to scale the regression prediction values between [0, 1]. The loss optimization function of this module is the Mean Squared Error (MSE). The formula for MSE is

(7) $$L_{MSE}={\sum_{i=0}^{m}\left(y_{i}-{\hat{y}}_{i}\right)}^{2}$$

Among them, *y* is the true IC50 value of the *i*-th data in the training set and *y* is the predicted value corresponding to the sample. The calculation formula of the total loss function of the entire model is

(8) $$L_{Total}=L_{MSE}+L$$

Generally, our deep transfer model consists of two parts. The first part is feature extraction, which reduces the dimensionality of gene expression data using stacking autoencoders and extracts the genetic features. The second part is domain adaptation, which approximates the feature distribution by maximizing the error between the source domain and the target domain. Finally, a regression task is used to predict continuous drug sensitivity values.

Next is model training and hyperparameter settings. Before model training, the input data of the two datasets are min-max normalization processing. The activation function of all layers of the feedforward neural network is RELU and its calculation formula is R(x)=max(0,x). The experiment uses version 1.15 of the deep learning tool Tensorflow. The training optimization algorithm is Adam and the learning rate is 0.0001. The training batch size batch size is set to 128 and the total number of training rounds epoch is set to 15. Early stopping is set to 3; that is, if the model’s loss does not decrease for 3 consecutive epochs, the training will stop.

#### 4.4.2. Deep Transfer Models Based on Autoencoders and Difference Metrics

**Figure 8:**
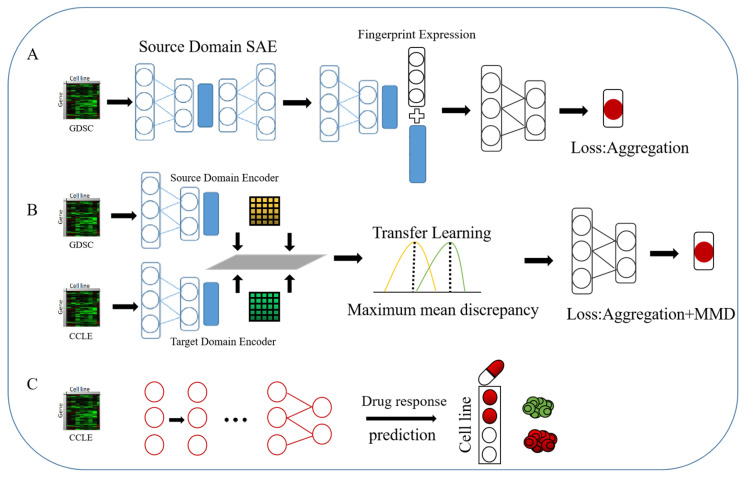
Flowchart of DADSP-B, which consists of two stacked autoencoders and a regression predictor. Part (**A**) uses only the source domain dataset GDSC through the feature extractor and regressor. Part (**B**) is the module of transfer learning, which minimizes the MMD loss of the gene features from the source domain and the target domain through the hidden layer features after the feature extractor to achieve a close feature distance between the two domains. The loss of the overall training is the regression loss of the MSE and MMD losses of the common training. Part (**C**) is the test part, which uses only the target domain dataset CCLE to predict drug sensitivity on the multitrained model.

We also propose a novel structural deep transfer learning model based on autoencoders mapped with the mean maximum discrepancy (MMD), which is also applied to the drug sensitivity prediction task. The MMD loss [87] is widely used in deep transfer learning [69,88]. DAN [69] is applied to minimize the difference between the fully connected layers of source and target domain features after the feature extractor. Our DADSP-B is illustrated in Figure 8. The feature extractor is consistent with that in DADSP-A. The stacked autoencoder is selected and the entire network is constructed by many feedforward networks.

(9) $$MMD\left(X^{S},X^{T}\right)=\|{\frac{1}{n^{S}}\sum_{i=1}^{n^{S}}\Phi \left(X_{i}^{S}\right)-\frac{1}{n^{T}}\sum_{j=1}^{n^{T}}\Phi \left(X_{j}^{T}\right)\|}^{2}$$

where *n* is the dimension of the data sample distribution; $X^{S}$ and $X^{T}$ are the sample distributions from the source domain and the target domain, respectively; and $\Phi \left(\cdot \right)$ maps the distribution of the original space to a regenerated Hilbert space and then measures the distance between the two sample distributions in the high-dimensional space.

During training, we adopt a freezing strategy [89]. The process is mainly divided into the following steps:

1. The feature extractor and regressor are trained using the source domain data to achieve the best possible performance for the source domain data on the regression task;
2. The target domain data are input into another stacked autoencoder [90]. We share the encoder parameters of the source domain data under the condition of freezing the regressor parameters, the reconstruction loss of the training target domain data layer by layer and the source domain data of the MMD loss.
3. Overall fine-tuning, freezing of the feedforward parameters of all feature extraction layers, and training of the regressor are performed. The loss at this time is only the MSE loss.

Our proposed DADSP-B differs from the adversarial-based DADSP-A in its use of the MMD loss function to measure the difference between the source and target domains. The MMD loss is incorporated into the reconstruction loss training of the stacked autoencoder. The purpose is to make the target domain data approximate the abstract low-dimensional features of the source domain data while reducing the dimensionality to obtain a low-dimensional representation. Through the freezing method, the parameters of the model are focused on the training of each part of the task rather than on the joint training of multiple loss functions, as in multitask learning [91]. We also compare and discuss the two models. The selection of hyperparameters is carried out in two steps. Firstly, we perform pre-training on the model using the original domain dataset, and then we apply grid search to select the hyperparameters after transferring to the target domain. Based on the performance observed in the target domain, we adjust the hyperparameters used in the pre-training on the original domain. For specific parameter configurations, please refer to the accompanying code.

In general, we propose two deep transfer learning models. One is a domain adaptation method based on domain adversarial learning, which achieves the goal of reducing the distance between the features of the source and target domains by maximizing the cross-entropy loss of the features of the two domains. The other is a transfer learning method based on a difference metric, which directly makes the feature representations of the hidden layers of the two domains close together through the MMD loss. Both methods pass the data through a deep feedforward network after a feature extractor to predict the drug sensitivity score.

### 4.5. Performance Metrics

We use two metrics, namely, the root mean square error (*RMSE*) and coefficient of determination (*R*^2^), to measure the performance of the model. *RMSE* is a commonly used indicator to measure regression prediction models and is expressed in (4). It is more sensitive to abnormally large errors and is more suitable for drug sensitivity prediction tasks than the mean square error (MSE). The coefficient of determination *R*^2^, also known as the determination coefficient or goodness of fit, is a statistical measure used to evaluate the degree of fit of a regression model. It represents the proportion of the variance in the dependent variable that can be explained by the model. The values of *R*^2^ range from 0 to 1, where a value closer to 1 indicates a better fit of the model to the observed data. The calculation formula, as shown in (5), is often used in regression analysis to judge the degree of fit of a regression equation and is used as a standard to measure model quality.

(10) $$RMSE=\sqrt{\sum {\left(y_{i}-{\tilde{y}}_{i}\right)}^{2}/N}$$

(11) $$R^{2}=1-{\sum {\left(y_{i}-{\tilde{y}}_{i}^{r0}\right)}^{2}/\sum \left(y_{i}-{\tilde{y}}_{i}\right)}^{2}$$

where *N* is the size of the data; $y_{i}$ and ${\tilde{y}}_{i}$ are the label data for drug sensitivity prediction and the predicted value corresponding to the *i*-th input data, respectively; and $\bar{y}$ is the mean value of the target drug ${\tilde{y}}_{i}^{r0}=k{\bar{y}}_{i}$, where *k* is the slope, as defined in (6).

(12) $$k=\sum y_{i}{\tilde{y}}_{i}/\sum {\bar{y}}_{i}^{2}$$

## 5. Conclusions

In this study, we proposed a deep transfer learning model for drug sensitivity prediction which not only predicts drug responses in cancer cell lines but also uses transfer learning to provide solutions for data distribution differences between genomics databases. Our model was trained on the GDSC and CCLE datasets and was shown to achieve the goal of knowledge transfer from the source domain to the target domain. We combined the gene expression signature of the cell line and the chemical structural signature of the drug as input to the model and successfully predicted the IC50 values through a deep neural network. From the results, through the deep transfer learning method, the information of the two databases was combined because only information from a single database was used. At the same time, the model showed excellent results in predicting unknown missing drug response pairs. In conclusion, our model can not only successfully perform information transfer between the two databases but also be used for real drug sensitivity prediction research.

From the performance comparison test, it can be seen that the feature extractor in the DADSP model is the cornerstone of the entire model performance results, so exploring better feature extraction methods for gene expression data and drug data will definitely improve the basic performance of the model. For data, source domain data and target domain data also have inconsistent label spaces. In this work, the labels are normalized. Therefore, in future work, we plan to add a label distribution optimization scheme based on DADSP to achieve more reliable domain adaptation.

## Figures and Tables

**Table 1:** Model comparison results.

Method	*RMSE*	*R* ^2^
DADSP-A	0.64	0.43
DADSPA-	0.71	0.31
DADSP-B	0.69	0.35
DeepDSC-1	0.82	0.11
DeepDSC-2	0.72	0.29
SLA	0.82	0.10
RF	0.75	0.27
LR	0.75	0.26
SVR	0.73	0.29

**Table 2:** Blind test results.

Method	*RMSE*	*R* ^2^
DADSP-A	0.69	0.32
DADSP-B	0.92	0.01
DeepDSC-1	0.70	0.29
SLA	0.72	0.30

**Table 3:** Comparison results of drug feature extraction methods.

Method	*RMSE*	*R* ^2^
DADSP-A	0.64	0.43
DADSP-A + SSP	0.67	0.35
DADSP-A + CNN	0.76	0.29
DADSP-A + GCN	0.74	0.23

**Table 4:** Critical genes in MKN7, ZR-75-30, and MEL-HO identified by the integrated gradient method.

MKN7		ZR-75-30		MEL-HO	
Critical Gene	Score	Critical Gene	Score	Critical Gene	Score
ENSG00000205364	0.002803	ENSG00000111700	0.003027	ENSG00000205364	0.003027
ENSG00000187908	0.002338	ENSG00000183032	0.002608	ENSG00000164821	0.002608
ENSG00000164821	0.002276	ENSG00000158023	0.002451	ENSG00000158023	0.002451
ENSG00000174469	0.002231	ENSG00000103316	0.002122	ENSG00000187908	0.002122
ENSG00000158023	0.002206	ENSG00000183668	0.002003	ENSG00000183032	0.002003
ENSG00000111404	0.002066	ENSG00000111249	0.001953	ENSG00000110077	0.001953
ENSG00000183032	0.002022	ENSG00000187908	0.001919	ENSG00000111404	0.001919
ENSG00000183668	0.001981	ENSG00000165168	0.001918	ENSG00000183668	0.001918
ENSG00000167083	0.001887	ENSG00000164821	0.001834	ENSG00000166049	0.001834
ENSG00000120162	0.001884	ENSG00000166049	0.001831	ENSG00000111249	0.001831

## Data Availability

Data are contained within the article.
